# Advanced 1,1-carboboration reactions with pentafluorophenylboranes

**DOI:** 10.1039/c5sc03282b

**Published:** 2015-10-08

**Authors:** Gerald Kehr, Gerhard Erker

**Affiliations:** a Organisch-Chemisches Institut , Universität Münster , Corrensstraße 40 , D-48149 Münster , Germany . Email: erker@uni-muenster.de

## Abstract

The 1,1 carboboration reaction of a variety of metal-substituted alkynes with simple trialkylboranes R_3_B yields the respective alkenylboranes (Wrackmeyer reaction). The use of the strongly electrophilic R-B(C_6_F_5_)_2_ reagents allows for much milder reaction conditions and gives good yields of the respective bulky alkenylboranes from conventional terminal alkynes by means of 1,2-hydride migration. Even internal alkynes undergo 1,1-carboboration with the R-B(C_6_F_5_)_2_ reagents, in this case yielding alkenylboranes by means of C–C bond cleavage. Phosphorus, sulfur or even boron containing substituents can serve as the migrating alkynyl substituents in the advanced 1,1-carboboration reactions using the R-B(C_6_F_5_)_2_ reagents. Sequential 1,1-carboboration of geminal bis(alkynyl) derivatives of these elements with the R-B(C_6_F_5_)_2_ boranes yields boryl substituted phospholes, thiophenes or even boroles in quite a variety. Vicinal bis(alkynyl)arenes or heteroarene substrates undergo benzannulation reactions in this way. Many of the -B(C_6_F_5_)_2_ substituted 1,1-carboboration products can be used as reagents in cross coupling reactions. A recently disclosed organometallic analogue, namely a 1,1-carbozirconation reaction is described.

## Abstract 2

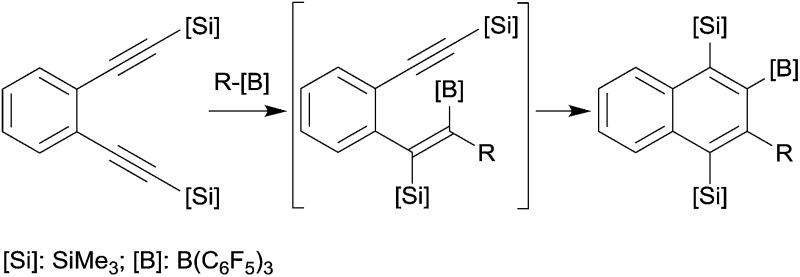
The advanced 1,1-carboboration reaction converts alkynes to alkenylboranes and allows a variety of sequential ring forming reactions of bis(alkynyl) compounds.

## Introduction: the Wrackmeyer reaction

**Scheme 1:**
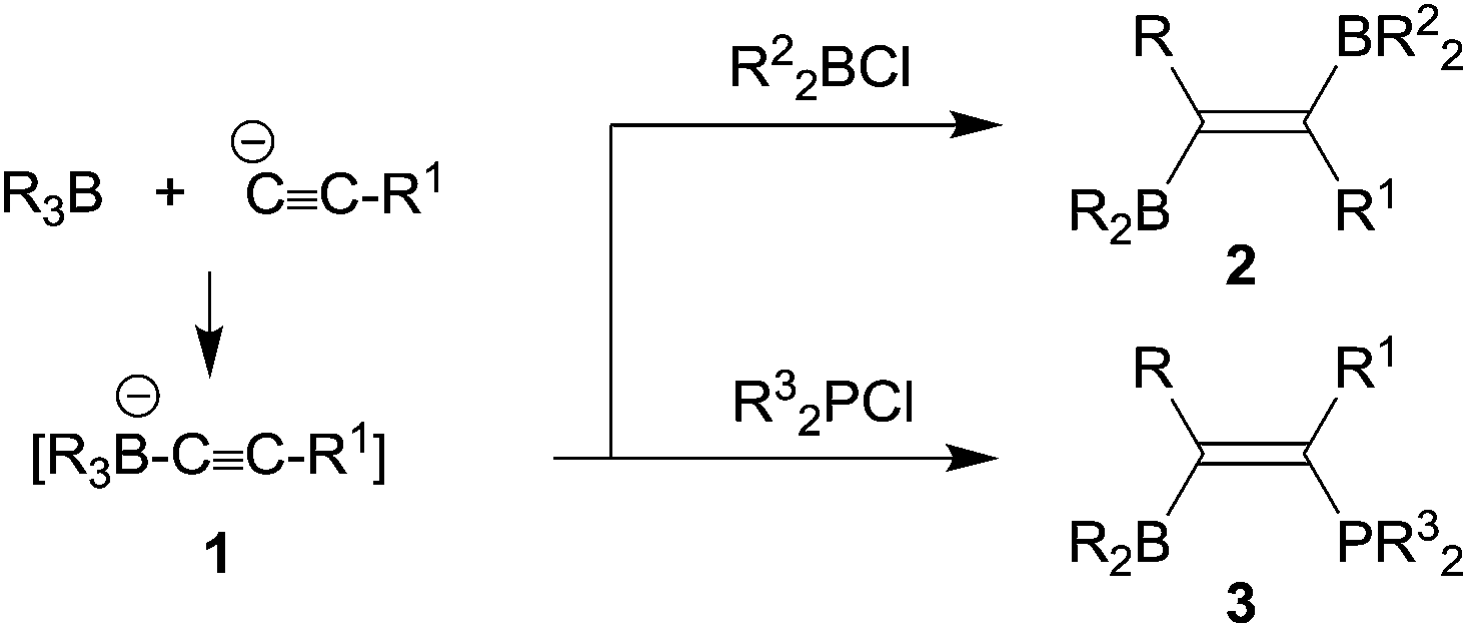


In the mid-1960s Binger and Köster described the reaction of alkynylborate anions **1** with electrophiles, *e.g.* R_2_BCl, to give unusual alkenylboranes **2**, that were formed by a reaction pathway involving 1,2-alkyl migration from the borate boron atom to the acetylenic α-carbon atom (see Scheme 1).^1^ The analogous reactions with R^3^_2_PCl reagents led to phosphanyl substituted alkenylboranes **3**.^2^ Since the alkynylborates had been prepared by alkynyl anion addition to the respective boranes, these reactions can be looked at as first, although quite specific examples of 1,1-carboboration reactions.^3^

**Scheme 2:**
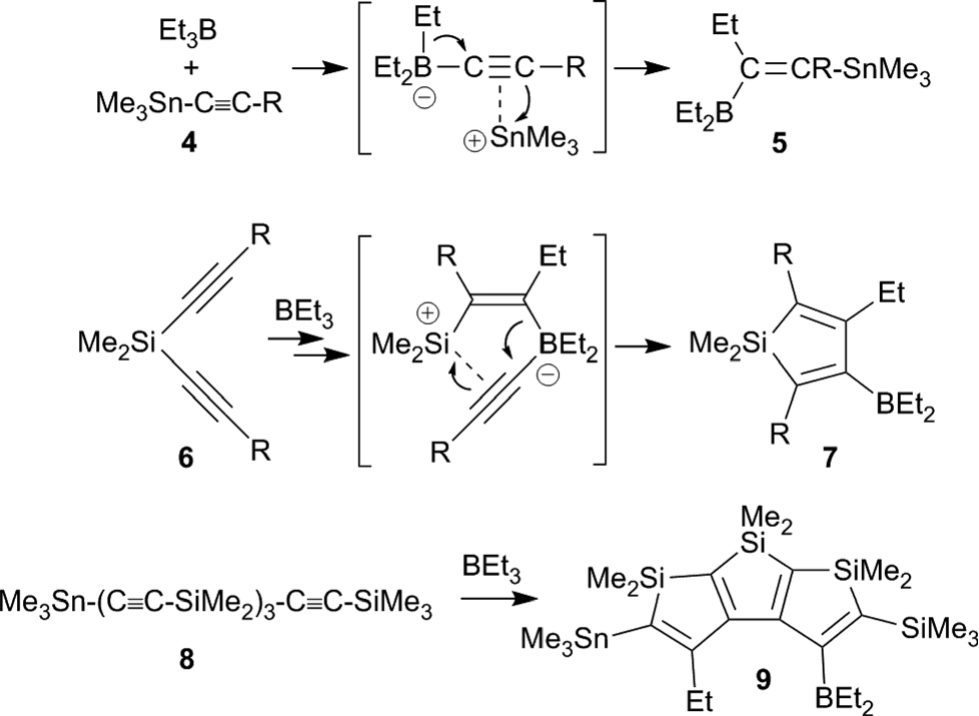


Wrackmeyer *et al.* developed this into a very useful neutral 1,1-carboboration variant (the “Wrackmeyer reaction”).^4^ They had found that *e.g.* trimethylstannyl-acetylenes **4** reacted rapidly with *e.g.* triethylborane to give the tetra-substituted alkenylboranes **5** (see Scheme 2).^5^ They found out that this reaction required a good metal containing migrating group at the alkyne substrate and showed that R_3_Ge- and R_3_Pb- (and some transition metal derived groups) were even better.^4,6^ Many examples using silyl substituents were used, although in these cases more forcing reaction conditions were often necessary.^7^ Intermediate metal/alkyne π-complexes were identified as reactive intermediates and even in a few cases characterized by X-ray diffraction.^4,8^ Subsequent developments carried out by the Wrackmeyer group involved silole^9^ and stannole syntheses^8c,d,10,11^ starting from the respective geminal bis(alkynyl) metal derivatives. A recent highlight of this development was the straightforward preparation of fused polycyclic silole systems such as compound **9** reported by Wrackmeyer *et al.* ^12^

**Scheme 3:**
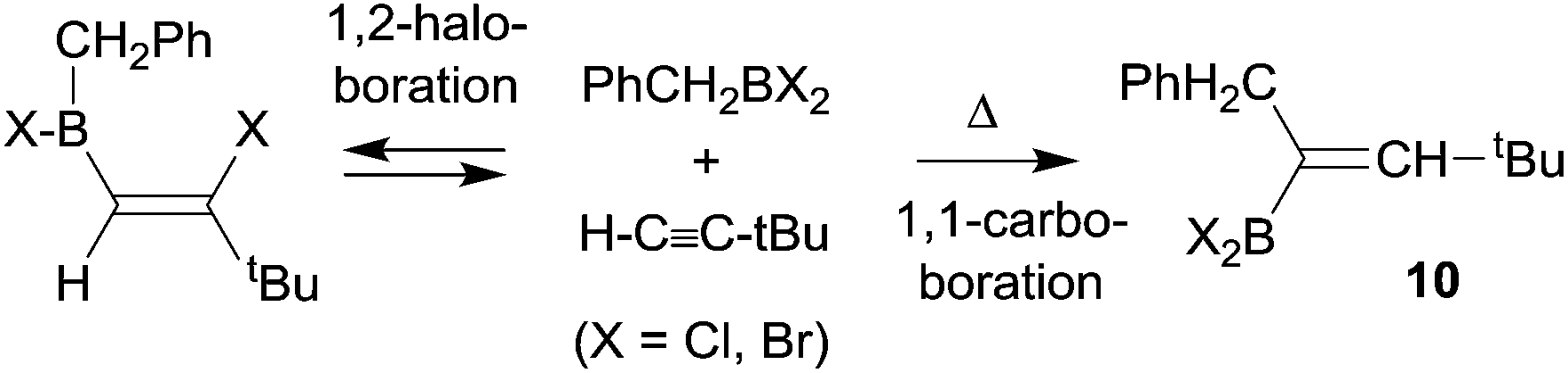


Paetzold *et al.* had described a few early experiments showing that 1,1-carboboration reactions could be accomplished in the absence of good metal containing migrating groups, although those reactions required high temperatures and the use of more strongly electrophilic dihalogenoboranes. A typical example is the formation of the alkenylborane **10** from benzyl-BBr_2_ with *tert*-butylacetylene (in this case H is migrating along the alkynyl framework) at 150 °C to give **10** (see Scheme 3). The products were actually identified by their oxidative deborylation products.^13^

## The advent of 1,1-carboboration with R-B(C_6_F_5_)_2_ reagents

**Scheme 4:**
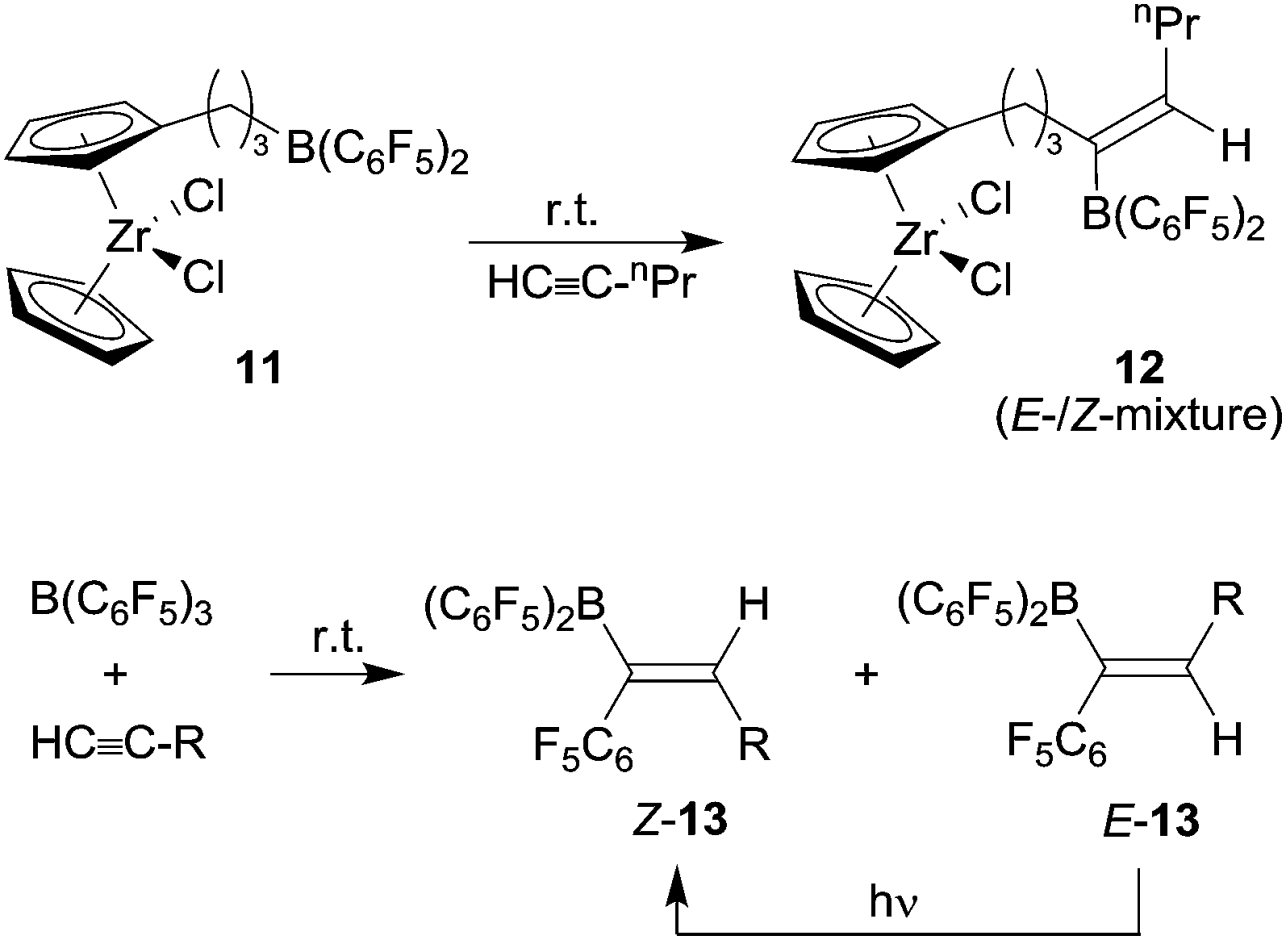


Paetzold's examples had indicated that the use of more Lewis acidic boranes might be useful for the development of 1,1-carboboration chemistry. This was realized by turning to B(C_6_F_5_)_3_ ^14^ and the related R-B(C_6_F_5_)_2_ reagents which in turn were often readily available by hydroboration routes using Piers' borane [HB(C_6_F_5_)_2_].^15^ We had found that the terminal alkyne 1-pentyne underwent selective 1,1-carboboration with the bis(pentafluorophenyl) substituted metallocene system **11** to give a *E*-/*Z*-mixture of the product **12** (see Scheme 4).^16^ The substituted alkyl group at boron migrated selectively in this process. We also reacted 1-pentyne with B(C_6_F_5_)_3_ under mild conditions and an *E*-/*Z*-mixture of the 1,1-carboboration products **13** was formed by C_6_F_5_ migration. In both examples subsequent *E*- to *Z*-alkenylborane isomerization was achieved by photolysis.

At about the same time Berke *et al.* reported the 1,1-carboboration reaction of phenylacetylene with B(C_6_F_5_)_3_ to give the *E*-/*Z*-mixture of the products **13** (R: Ph) (see Scheme 4).^17^ From these early examples we subsequently developed the 1,1-carboboration reaction of alkynes with R-B(C_6_F_5_)_2_ reagents into a viable alternative to the ubiquitous hydroboration route to alkenylboranes.^18^ We used the products as reagents in cross-coupling reactions^18^ as well as borane Lewis acid components in catalytic metal-free frustrated Lewis pair hydrogenation reactions.^19^ Most of this and related work has been reviewed by us,^20^ so this will not be repeated here.

**Scheme 5:**
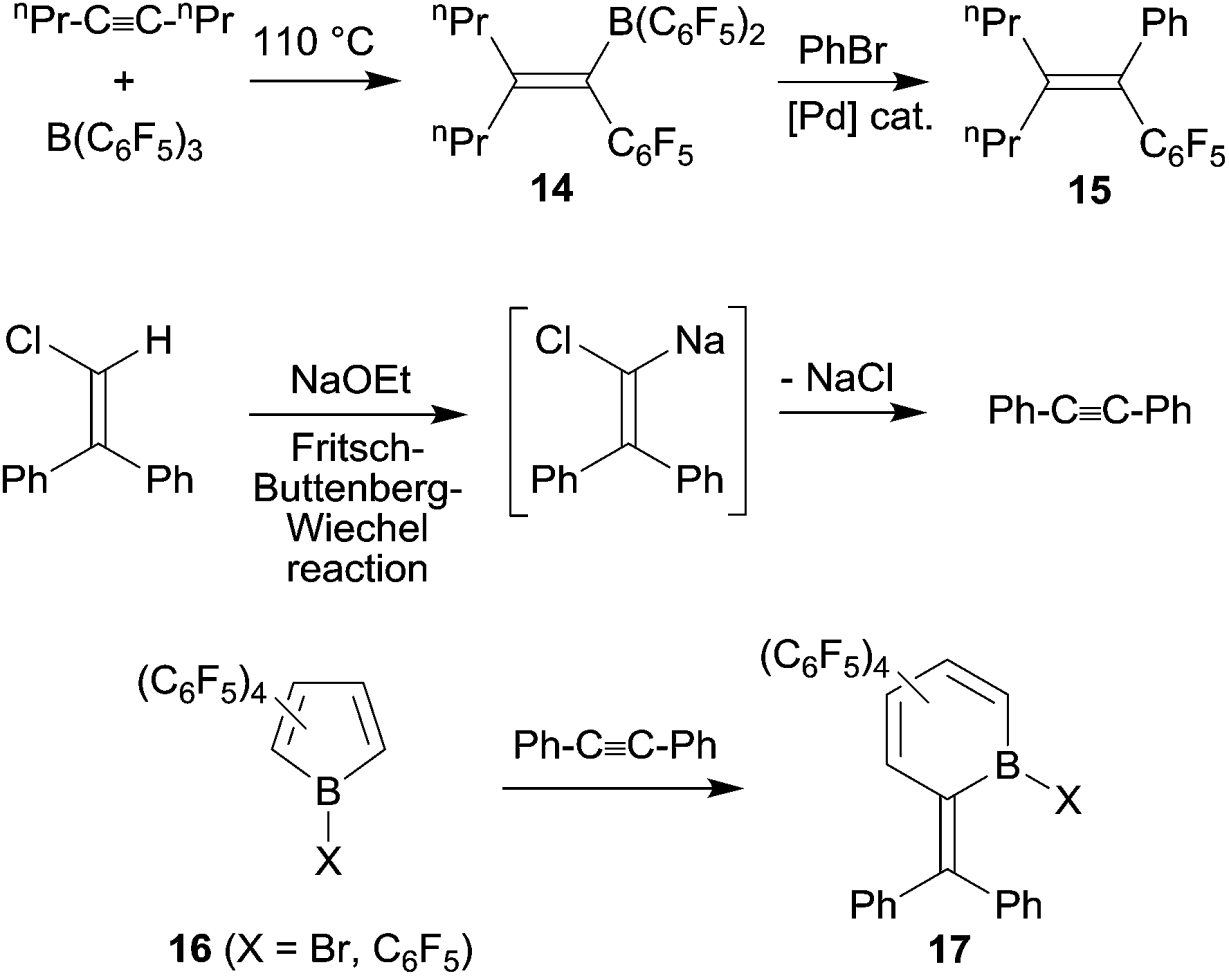


Under slightly more forcing conditions the 1,1-carboboration reaction could even be used for C–C bond activation reactions. Cleavage of unactivated carbon–carbon bonds is still relatively rare, it is mostly achieved using metal complex initiation or catalysis.^21^ 1,1-Carboboration in some cases allows to achieve a metal-free cleavage of non-activated sp-carbon-sp^2^ (or sp^3^)-carbon-σ-bonds. The reaction of 4-octyne with B(C_6_F_5_)_3_ is a typical example: at 110 °C the reaction proceeds cleanly by C–C bond cleavage and 1,2-migration of the *n*-propyl group along the alkynyl framework to give **14** (see Scheme 5). Compound **14** was subsequently converted by a Pd-catalyzed cross coupling reaction to the boron free product **15**.^22^ In a way this 1,1-carboboration reaction resembles the reverse of a Fritsch–Buttenberg–Wiechel-rearrangement, although the reaction mechanisms of these two reactions are not related.^23^ Piers *et al.* provided nice examples of 1,1-carboboration reactions at reactive C_6_F_5_-substituted borole frameworks with tolane that also proceeded with C–C bond cleavage (see Scheme 5).^24^

A strength of the original Wrackmeyer reaction had been the formation of unsaturated cyclic compounds (*e.g.* siloles, see Scheme 2) from di- and oligoacetylene precursors. This has been introduced into the reaction schemes of the B(C_6_F_5_)_2_ based “advanced 1,1-carboboration” reaction protocols, which has resulted in a variety of remarkable developments. Some of these will be briefly highlighted in the following chapters of this perspective.

## Benzannulation

**Scheme 6:**
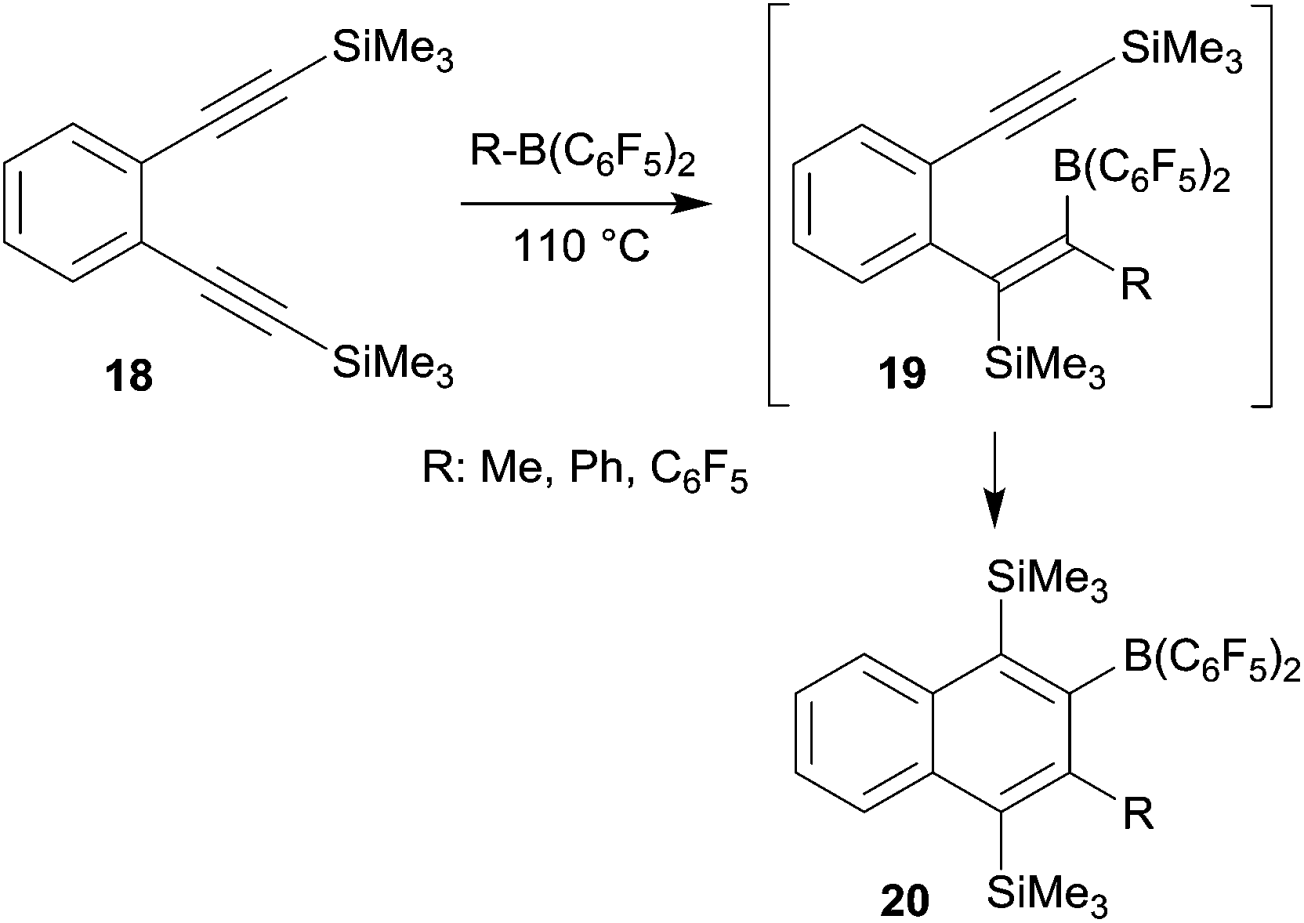


*o*-Bis(alkynyl)benzenes have been used as the starting materials for the synthesis of naphthalene derivates by a sequence of consecutive 1,1-carboboration reactions. This application of the advanced carboboration reaction contributes an interesting addition to the existing repertoire of benzannulation reactions of 1,2-bis(alkynyl)benzenes.^25^ We have shown that the readily available systems **18** reacted smoothly with a small series of R–B(C_6_F_5_)_2_ reagents to form the naphthalenes **20**, probably in a two-step reaction sequence proceeding *via* the intermediate **19** (see Scheme 6). The B(C_6_F_5_)_2_ substituent of product **20** was subsequently utilized for cross-coupling reactions and the trimethylsilyl-substituents could conveniently be removed.^26^

**Scheme 7:**
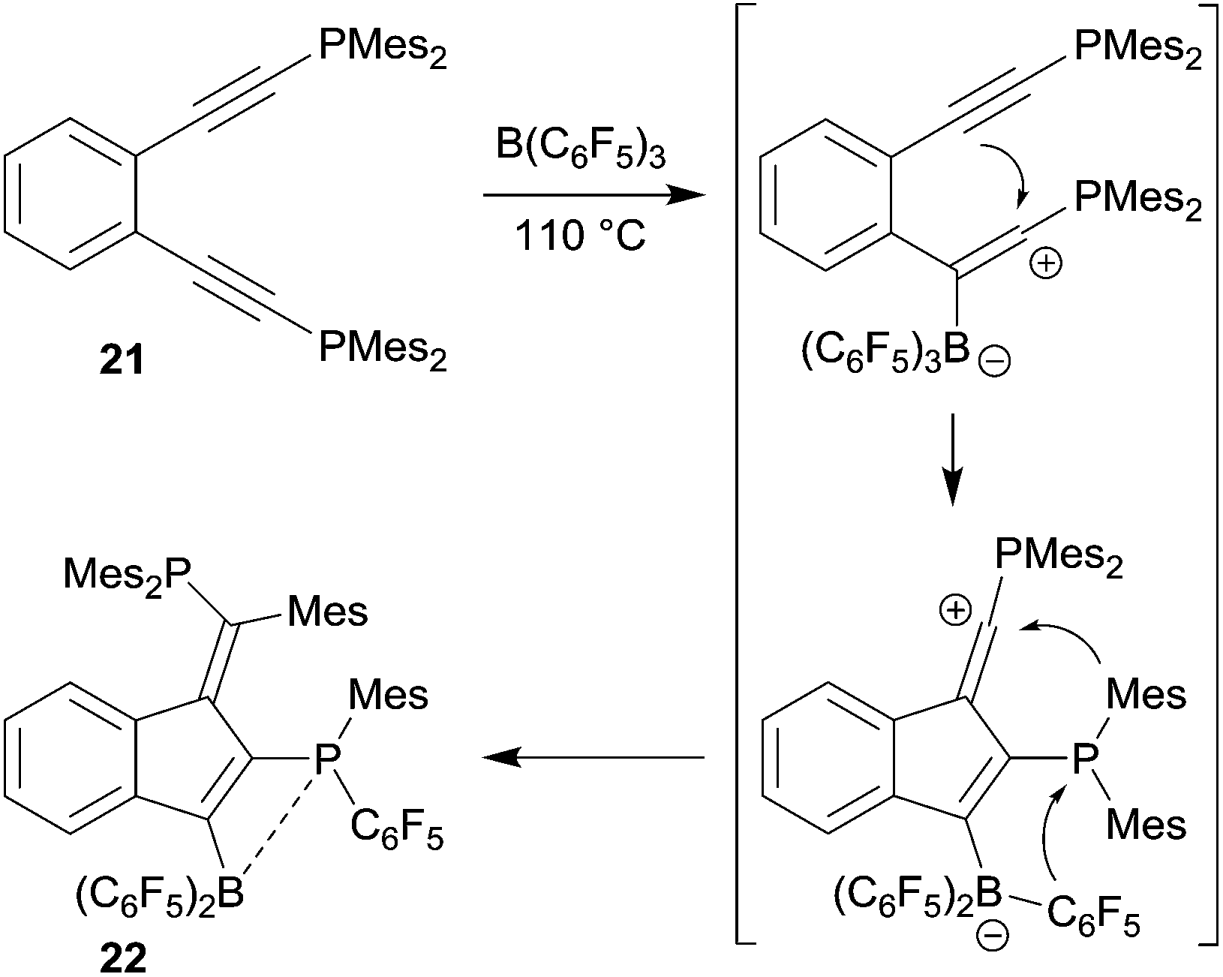


The reaction of *o*-bis(Mes_2_P-ethynyl)benzene (**21**) with B(C_6_F_5_)_3_ gave a different product type **22**. In this case we assumed that a competing pathway was favoured which represents the seldomly observed carbocation alternative to the 1,1-carboboration reaction (see Scheme 7).^27^ It may be that the ensuing formation of a B–P interaction favours this alternative pathway.

**Scheme 8:**
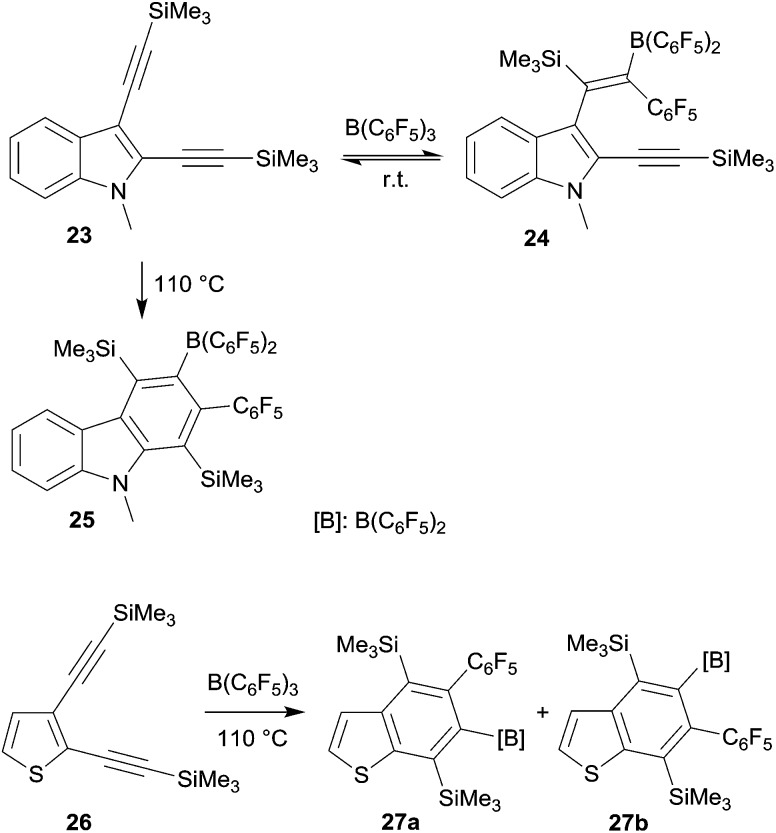


Benzannulated heterocycles are often prepared by forming the heterocyclic ring at the already present benzenoid aromatic ring. 1,1-Carboboration reactions allow for an alternative strategy. This could be shown in a variety of examples using the B(C_6_F_5_)_3_ borane reagent (see Scheme 8).^28^ Its reaction with the indole derivate **23** initially gave only a conventional single 1,1-carboboration product **24** at the distal alkynyl moiety. But this was apparently reversible to eventually cleanly yield the respective carbazole derivative **25** under thermodynamic control. In a similar way, the benzothiophenes **27a,b** and a functionalized quinoline were prepared by 1,1-carboboration of the respective 2,3-bis(alkynyl) heteroarenes.

## Five-membered heterocycles by 1,1-carboboration

**Scheme 9:**
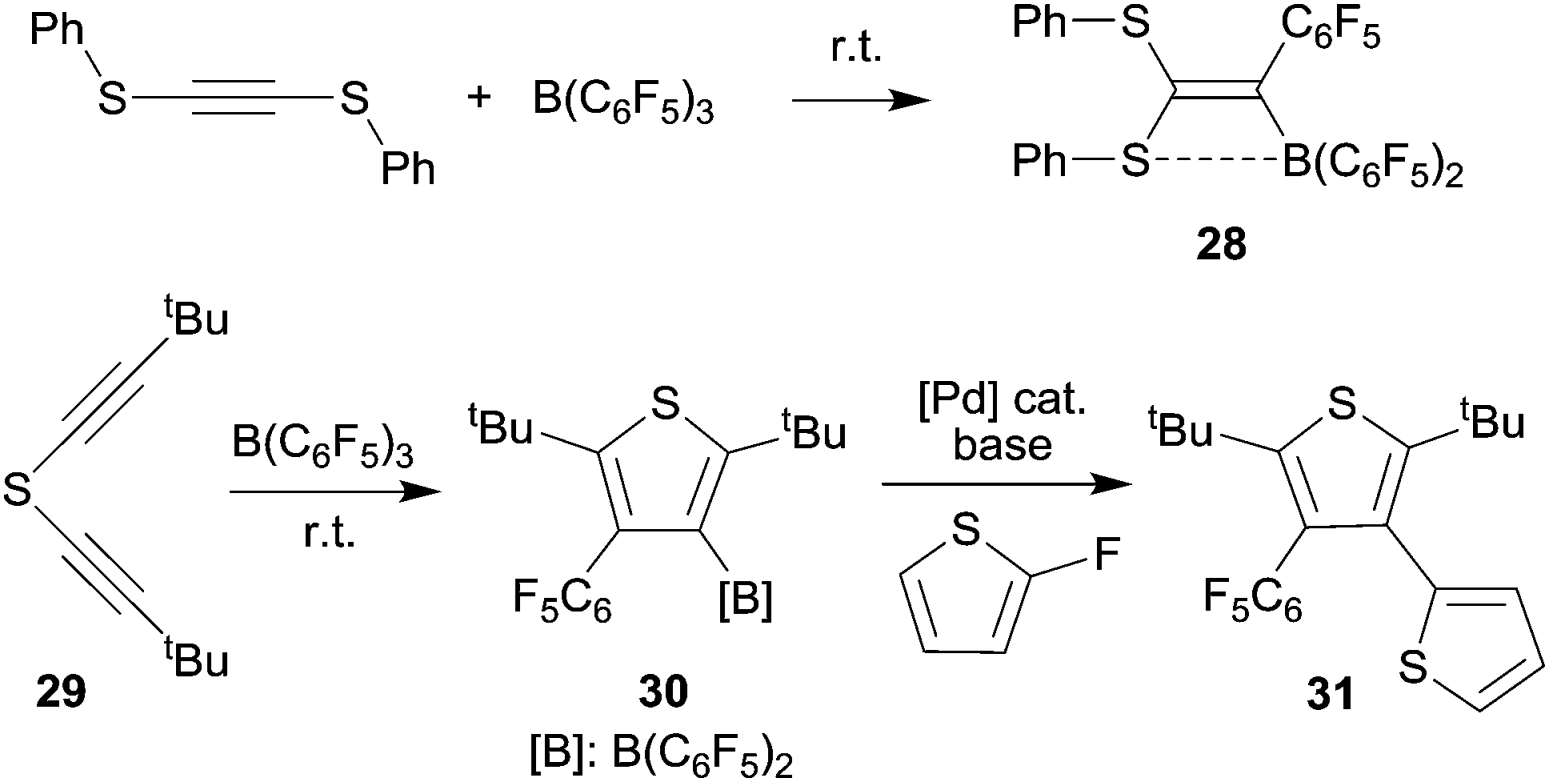


**Scheme 10:**
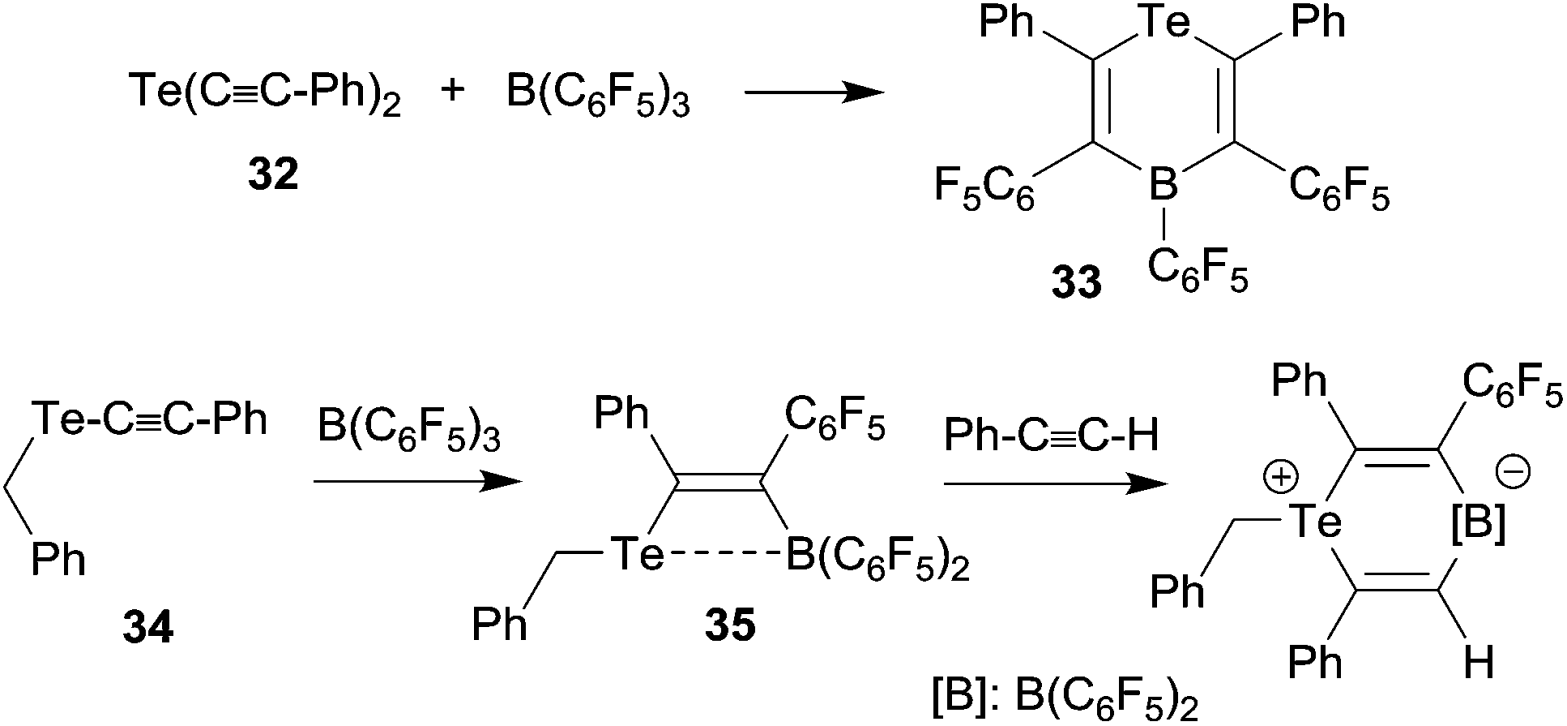


We have shown that thiolate substituents can serve as suitable migrating groups in 1,1-carboboration reactions (see Scheme 9 for typical examples).^29^ This effect was used to develop a sequential 1,1-carboboration route to a boryl substituted thiophene derivative. The product **30** was deborylated by treatment with acetic acid. It also served as a cross-coupling reagent for the synthesis *e.g.* of a substituted bithiophene derivative **31**. Stephan *et al.* showed that the closely related bis(alkynyl) tellurium compound **32** underwent a consecutive series of 1,1-carboboration reactions with *e.g.* B(C_6_F_5_)_3_ to form an example of less often observed six-membered heterocyclic ring systems (**33**) (see Scheme 10).^10,30^ A dimeric product was formed in a reversible competitive pathway.

The benzyl alkynyl telluride **34** undergoes 1,1-carboboration with B(C_6_F_5_)_3_ to form the Te/B frustrated Lewis pair (FLP) **35** that *e.g.* undergoes 1,2-addition to phenylacetylene (see Scheme 10).^31^

**Scheme 11:**
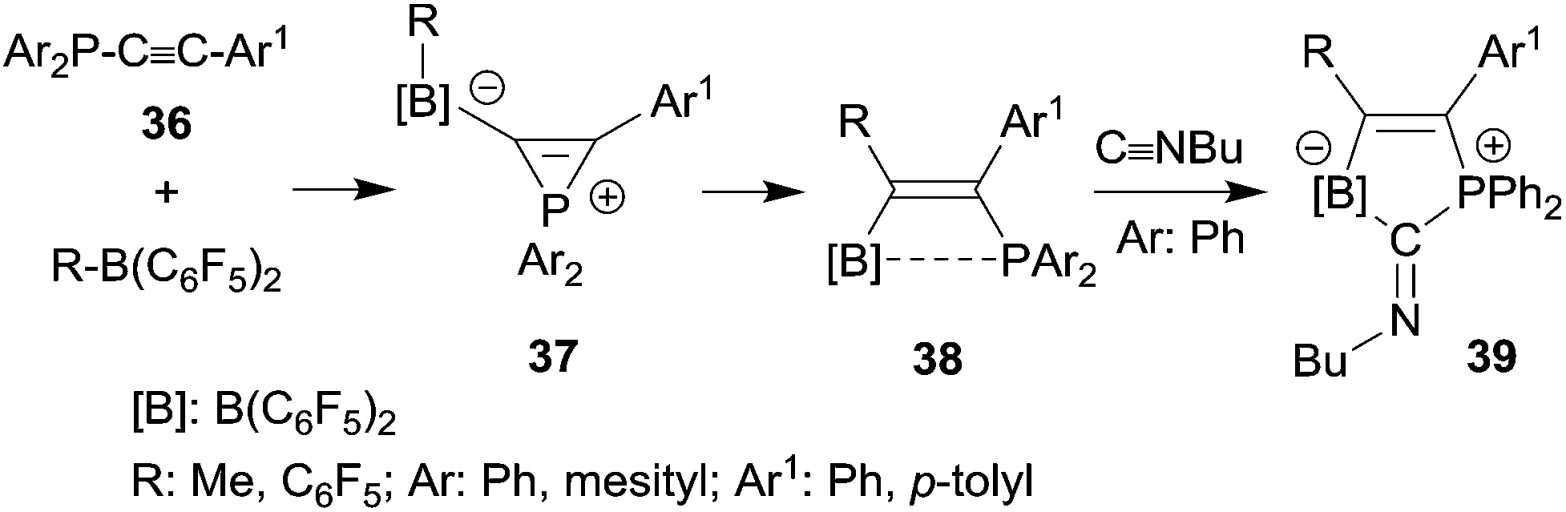


Phosphanyl-substituted alkynes **36** underwent facile 1,1-carboboration reactions with the strongly Lewis acidic R-B(C_6_F_5_)_2_ reagents (see Scheme 11). There is evidence that the reactions proceed *via* zwitterionic phosphirenium borate intermediates **37**, some of which were isolated under suitable reaction conditions and characterized by their very typical high field ^31^P NMR signals and by X-ray diffraction.^32,33^ The resulting P···B interacting Lewis pairs **38** ^32–34^ were rather unreactive, but several of them underwent a remarkable cooperative 1,1-addition reaction to isonitriles to give compounds **39**,^34^ a reaction of these bifunctional main group element compounds that is reminiscent of the coordination behaviour typical of transition metal complexes.^35^

**Scheme 12:**
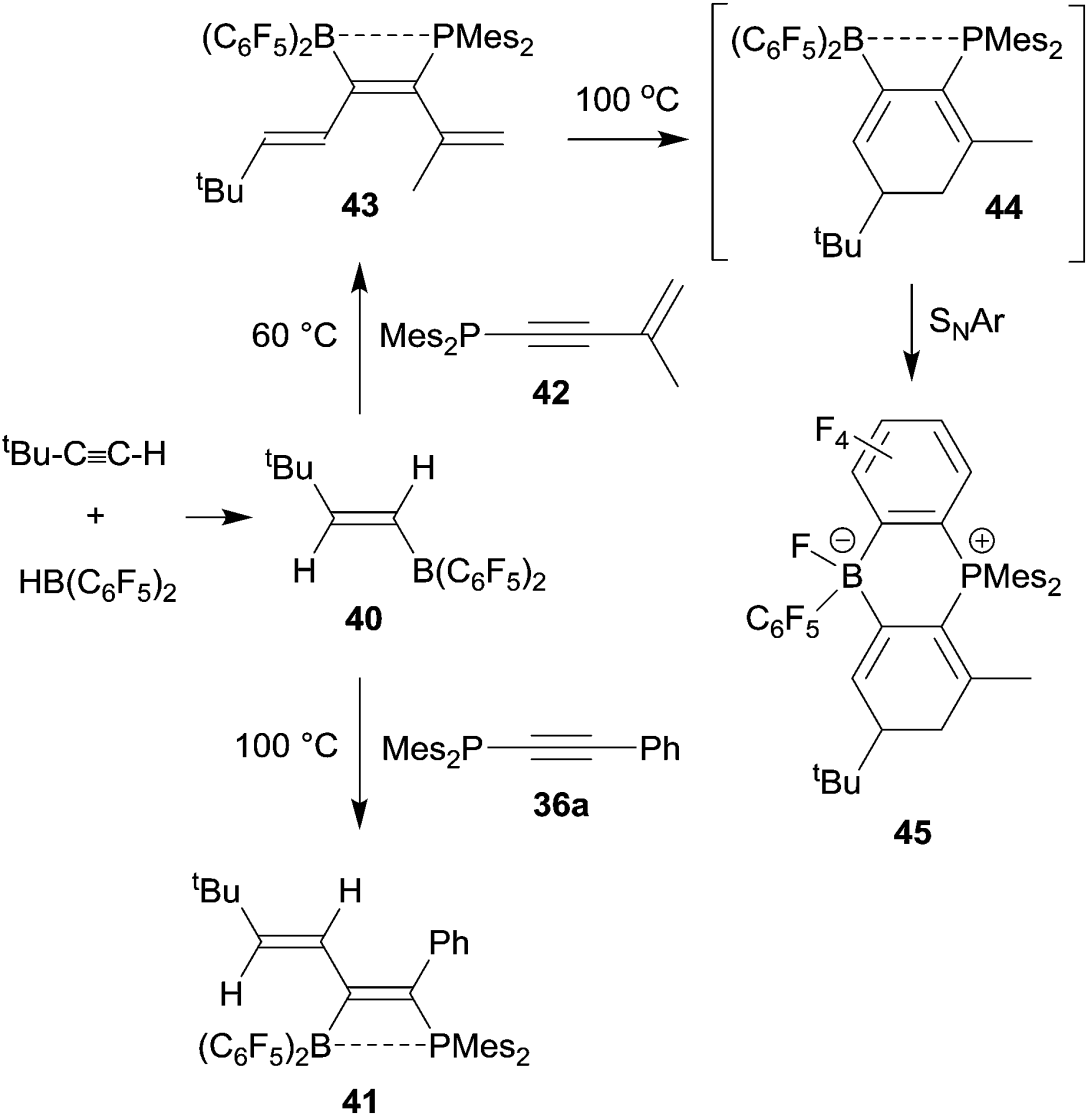


Phosphinoalkynes even undergo clean 1,1-carboboration reactions with alkenylboranes, provided their substitution pattern is not too sterically constrained. Thus, the *trans-tert* butylethenylborane **40**, prepared by hydroboration of *tert* butyl acetylene with Piers' borane, underwent a clean 1,1-carboboration reaction with the diarylphosphinoalkyne **36a** (Ar: mesityl, Ar^1^: Ph) to give the P/B functionalized conjugated diene product **41** (see Scheme 12).^36^ This reaction scheme can be extended: typically, the alkenylborane **40** reacted analogously with a variety of diarylphosphinoenynes (*e.g.* **42**) to give the respective hexatriene derivative **43** by 1,1 carboboration. Compound **43** eventually underwent electrocyclic ring closure upon heating to generate **44** which subsequently formed the product **45** by means of an intramolecular nucleophilic aromatic substitution reaction (see Scheme 12).^37^

**Scheme 13:**
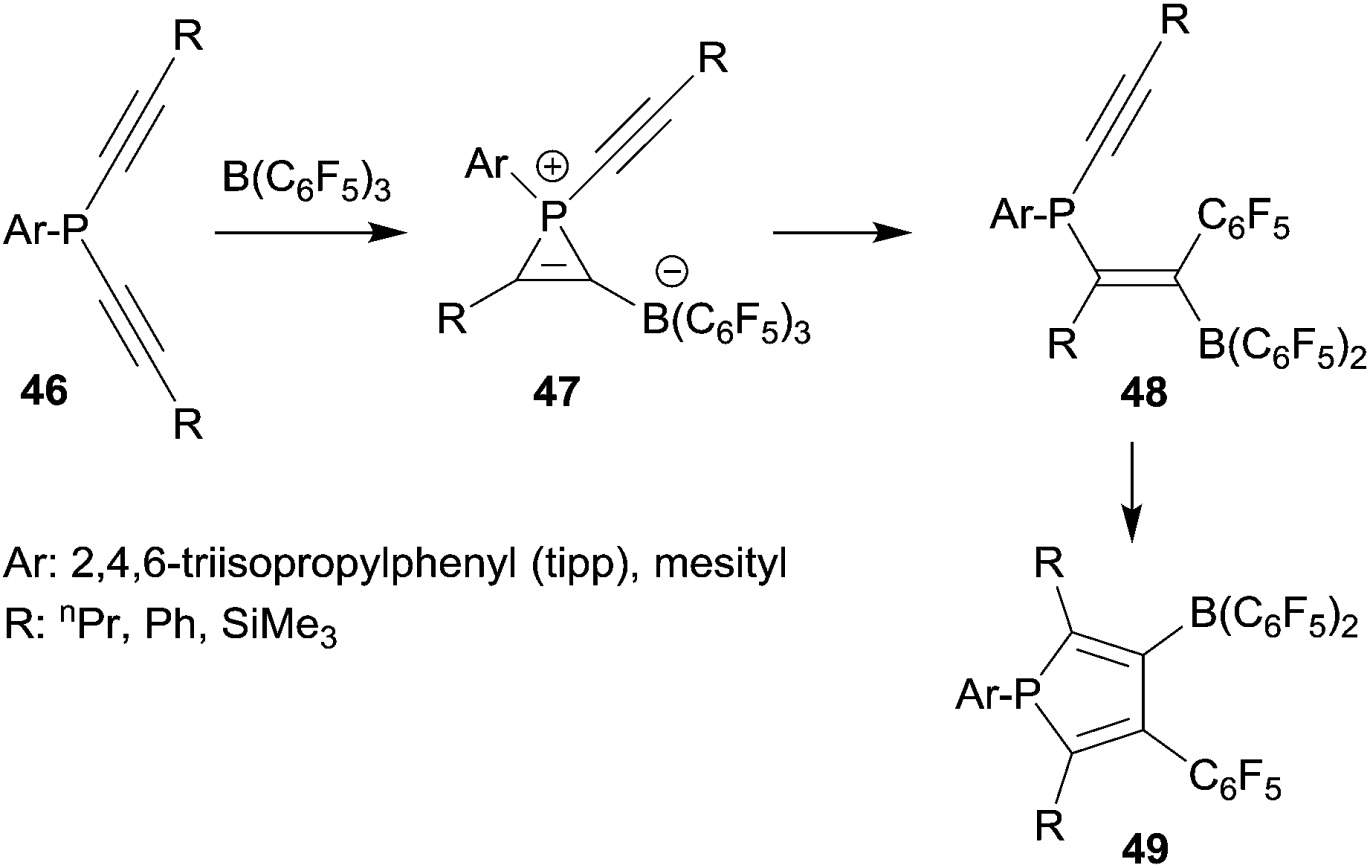


Since phosphanyl units turned out to be quite good migrating groups we developed these reactions into 1,1-carboboration phosphole syntheses. In a series of typical experiments we reacted several arylbis(alkynyl)phosphanes with B(C_6_F_5_)_3_. At slightly elevated temperatures the corresponding boryl substituted phospholes **49** were obtained in good yields (see Scheme 13).^38^ The reaction course was followed by temperature dependent ^31^P NMR spectroscopy. In this way we were able to observe some intermediates of the typical sequential 1,1-carboboration pathway along the way ranging from initial phosphane/borane adduct formation through the stages of the phosphirenium/borate zwitterion formation followed by the 1,1-carboboration product isomers at the first alkynyl unit, all the way to the final phosphole products. An example of the alkenylborane intermediates **48**, several phosphirenium/borates **47** and a variety of the final phosphole products were isolated and eventually characterized by X-ray diffraction.

**Scheme 14:**
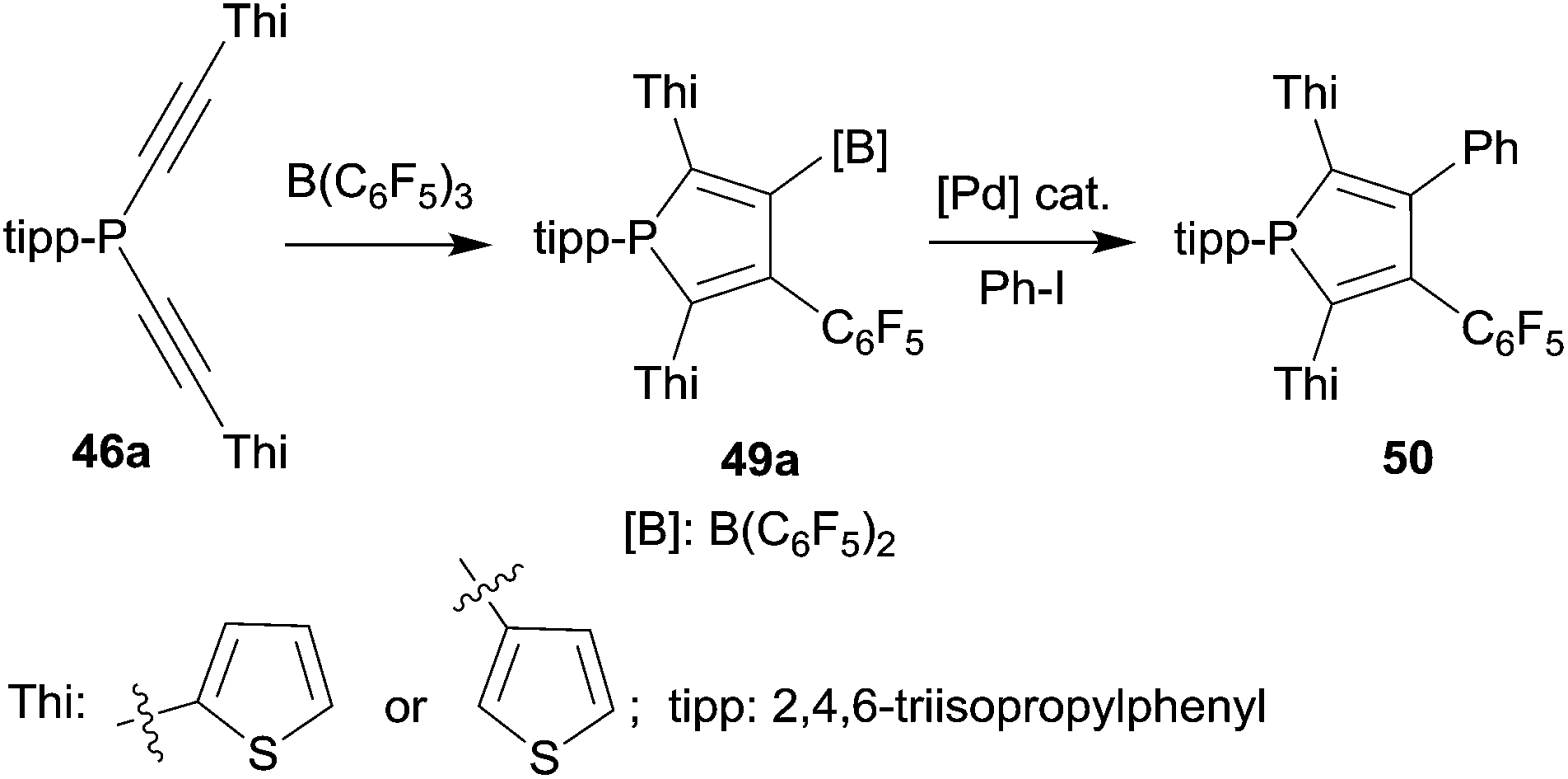


Phospholes with a variety of substituent patterns were prepared by the 1,1-carboboration route. Attachment of both the thienyl substituent isomers at the 2,5-positions was achieved *via* the respective thienyl substituted alkynyl-phosphane starting materials **46a** (see Scheme 14). The B(C_6_F_5_)_2_ functionality of the respective phosphole **49a** was later used for replacement by aryl groups by means of Pd-catalyzed cross-coupling. Scheme 14 shows typical examples. The photophysical properties of several of these phosphole derivatives (and of their corresponding phosphole-oxides) were investigated.^39^

**Scheme 15:**
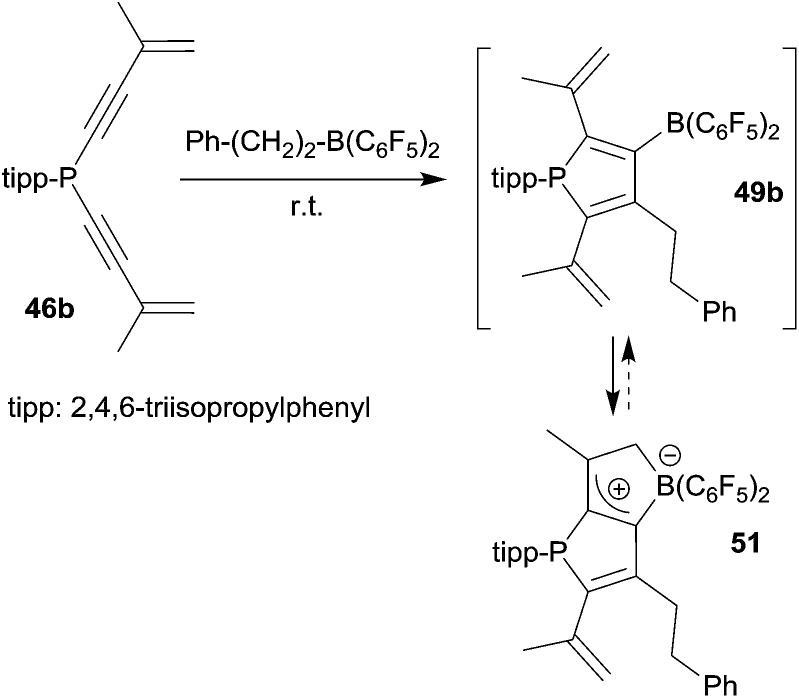


The alkenyl functionalized borylphosphole **49b**, which was formed by the 1,1-carboboration sequence of **46b** with the borane Ph–CH_2_CH_2_–B(C_6_F_5_)_2_ (and a few other RB(C_6_F_5_)_2_ reagents), showed a subsequent cyclization reaction to give the ring-closed isomer **51**, characterized by NMR spectroscopy and by X-ray diffraction (see Scheme 15).^40^ This reaction represents a rare example of a rapidly proceeding thermally induced bora-Nazarov reaction.

**Scheme 16:**
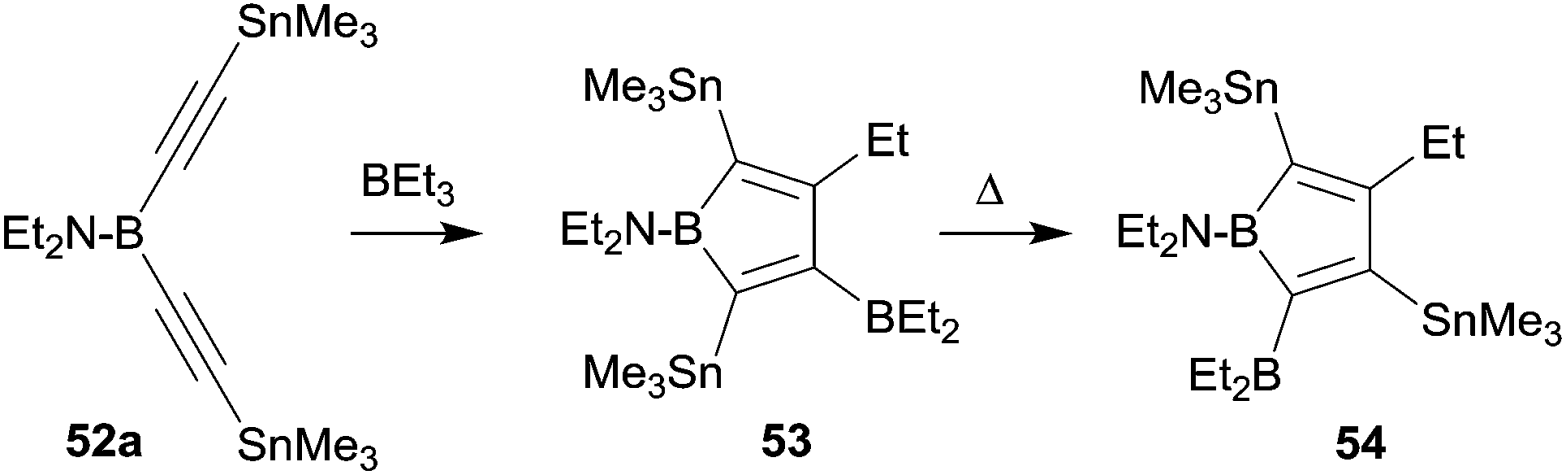


Boroles are formally antiaromatic compounds. They constitute 4π-electron systems that have a singlet ground state. The unsubstituted parent borole has only be synthetically obtained transition metal complex stabilized. Isolated borole examples are usually rather highly substituted. They are mostly prepared by reaction sequences involving metal–boron exchange reactions, often from the respective stannole which are available by means of transmetallation reactions from the corresponding dilithiobutadienes or the zirconacyclopentadienes.^41^ Wrackmeyer *et al.* had reported an early example of a borole synthesis by 1,1-carboboration using the superb ability of the SnMe_3_ substituent to serve as migrating group.^42^ Starting from **52a** they were able to generate the borole system **53**, which, however, rearranged at 60 °C to give **54**, the product that was eventually isolated as an oil (see Scheme 16).

**Scheme 17:**
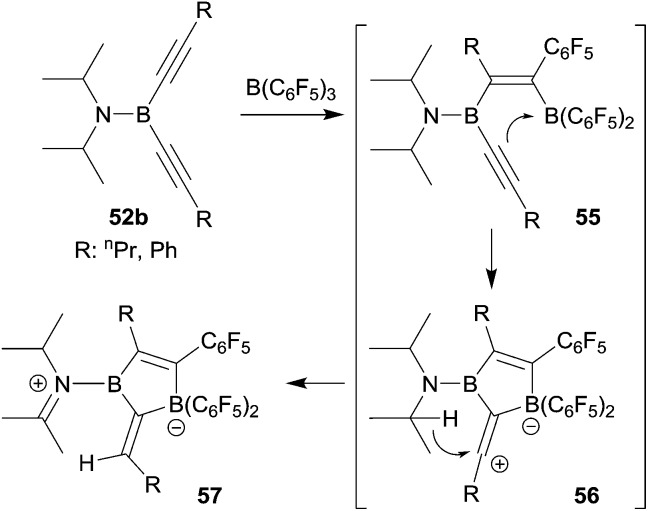


We investigated whether the borole formation by the typical 1,1-carboboration sequence starting from bis(alkynyl)boron compounds could be improved by using the strongly Lewis acidic R-B(C_6_F_5_)_2_ reagents. In a first attempt we treated the bis(isopropyl)amido boron acetylide **52b** with B(C_6_F_5_)_3_, but this did not lead to the formation of the anticipated borole product. Instead it seemed that the sometimes observed typical carbocation pathway took over after the first 1,1-carboboration step and we eventually isolated the alternative product **57** (see Scheme 17).^43^

**Scheme 18:**
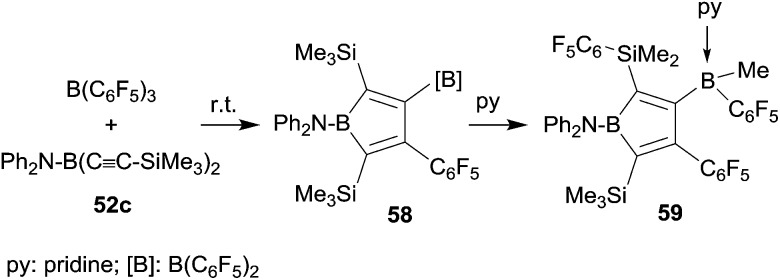


It was obvious that we should remove the hydride donor ability of the stabilizing substituent at the stage of the intermediate **56** and indeed the reaction of the corresponding diphenylamido substituted bis(alkynyl)borane **52c** with B(C_6_F_5_)_3_ gave the expected borole derivative **58**. Compound **58** was characterized spectroscopically. Its treatment with pyridine gave the pyridine addition product **59** of the pendant B(C_6_F_5_)_2_ functionality (see Scheme 18). In this step a substituent exchange reaction between the B(C_6_F_5_)_2_ group and the proximate SiMe_3_ substituent took place as it is sometimes observed in such situations (see above). Compound **59** was characterized by X-ray diffraction.^44^

**Scheme 19:**
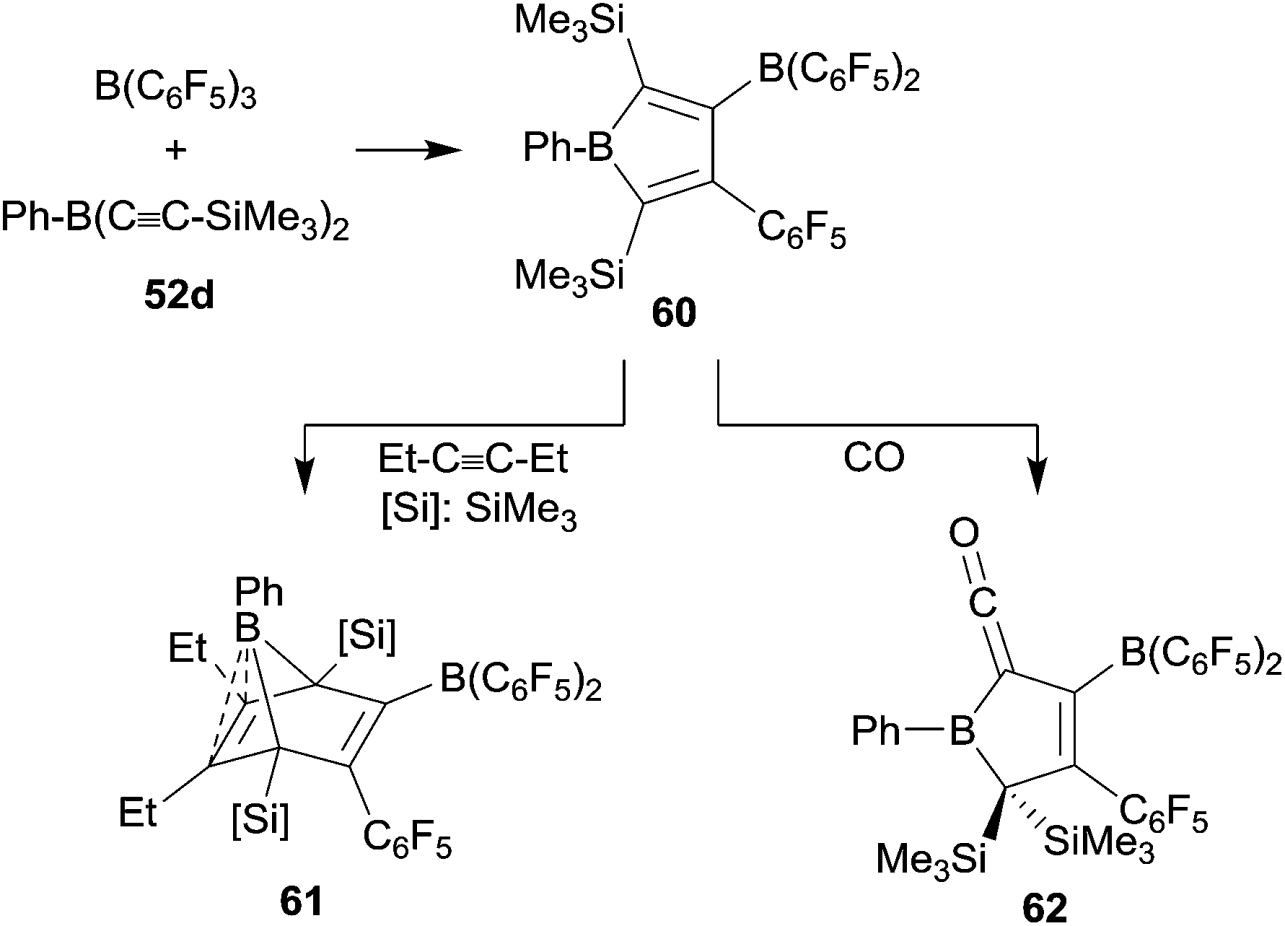


The phenylbis(alkynyl)borane **52d** reacted cleanly with B(C_6_F_5_)_3_ to give the borole **60** by sequential 1,1-carboboration. It was isolated in 43% yield and characterized by X-ray diffraction (see Scheme 19).^44^ Compound **60** underwent [4 + 2] cyclo-addition with 3-hexyne to give **61**. The electron-rich endocyclic C=C double bond in **61** shows a close contact to the apical boron atom, which gives a distorted square-pyramidal coordination geometry.^44,45^ A few [B]–H boranes had been shown to give borane carbonyls upon exposure to carbon monoxide.^46^ Piers *et al.* had shown that some pentaaryl boroles in a similar way form thermolabile [B]–CO adducts.^47^ We could show that the borole **60** reacts with carbon monoxide. We assume that initially a CO adduct at the borole boron atom was formed which then rapidly rearranged to yield the unusual ketenyl borane product **62** (see Scheme 19).^45^

**Scheme 20:**
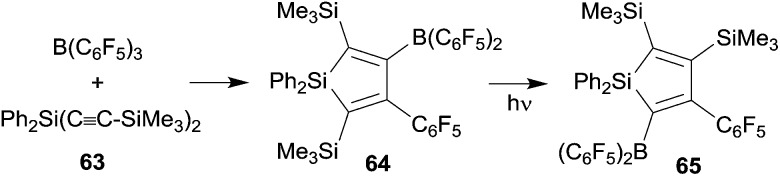


We have also prepared some silole derivatives by means of the R-B(C_6_F_5_)_2_ advanced 1,1-carboboration protocol although in this case the original Wrackmeyer procedure did not need much substantial improvement. However, silole formation by the reaction of the respective R_2_Si(C≡CR^1^)_2_ reagents with B(C_6_F_5_)_3_ proceeded under milder conditions, typically forming the products in high yields at room temperature.^20,48,49^ The silole **64** formed from **63** and B(C_6_F_5_)_3_ is a typical example. Interestingly the silole **64** underwent isomerization to **65** upon photolysis. This reaction might be regarded as an example of the silicon analogue of a di-π-methane rearrangement (see Scheme 20).^20,49^

## Functional group chemistry at frustrated Lewis pairs

**Scheme 21:**
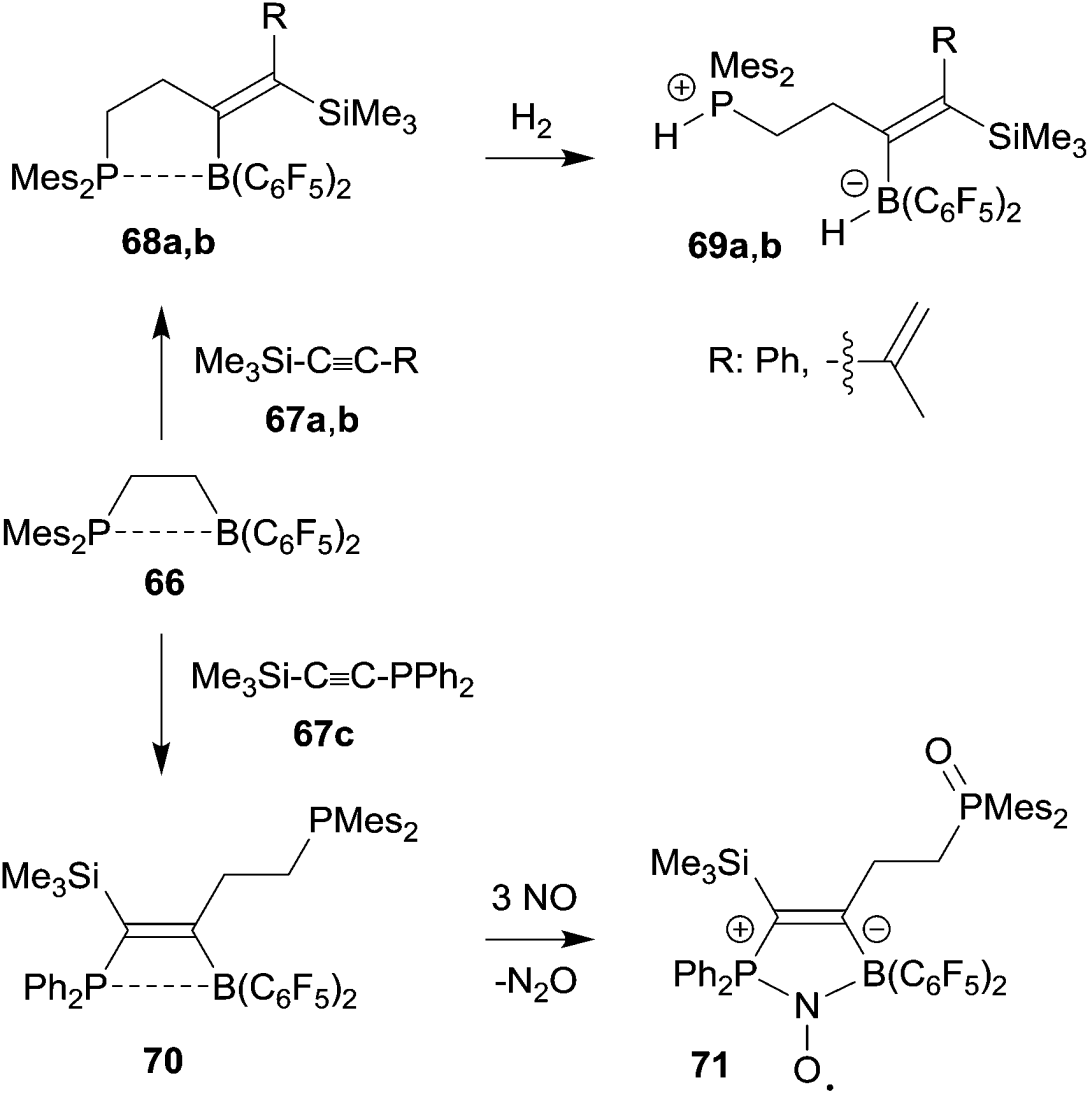


Frustrated Lewis pair (FLP) chemistry has seen a remarkable development, especially in view of the ability of metal free small molecule binding and activation by these main group element pairs of active Lewis acids and bases.^50^ Intramolecular FLPs have made a significant contribution to this chemistry. Most intramolecular FLPs were very conveniently made by hydroboration of alkenyl phosphanes or enamines with HB(C_6_F_5_)_2_. However, once prepared the high reactivity and sensitivity of especially the boron Lewis acid component has substantially limited any further modification of these systems. 1,1-Carboboration chemistry has provided some solution of this synthetic problem by selectively using the borane functionality for chemical FLP modification. Here are a few examples (see Scheme 21).

The ethylene-bridged intramolecular P/B FLP **66** undergoes a 1,1-carboboration reaction with trimethylsilylphenyl acetylene **67a** to give the expected C_3_-bridged P/B FLP **68a**. This features a weak phosphane/borane interaction. Nevertheless, the system **68a** is able to heterolytically cleave dihydrogen with formation of **69a** under mild conditions.^51^ The FLP **66** reacted in a similar way with the trimethylsilylenyne **67b** to give the diene containing FLP **68b** which is also a metal-free hydrogen activator.^52^ The 1,1-carboboration reaction of **66** with the phosphanyl substituted alkyne **67c** gave a bifunctional P/P/B FLP **70**. It reacted in very special way with nitric oxide (NO). First the pendant PMes_2_ group was oxidized and then NO cooperatively added to the remaining P/B FLP system to give the persistent P/B FLP NO radical **71**.^51^ It underwent H-atom abstraction reactions typical for this class of compounds.^53^

## 1,1-Carbozirconation

**Scheme 22:**
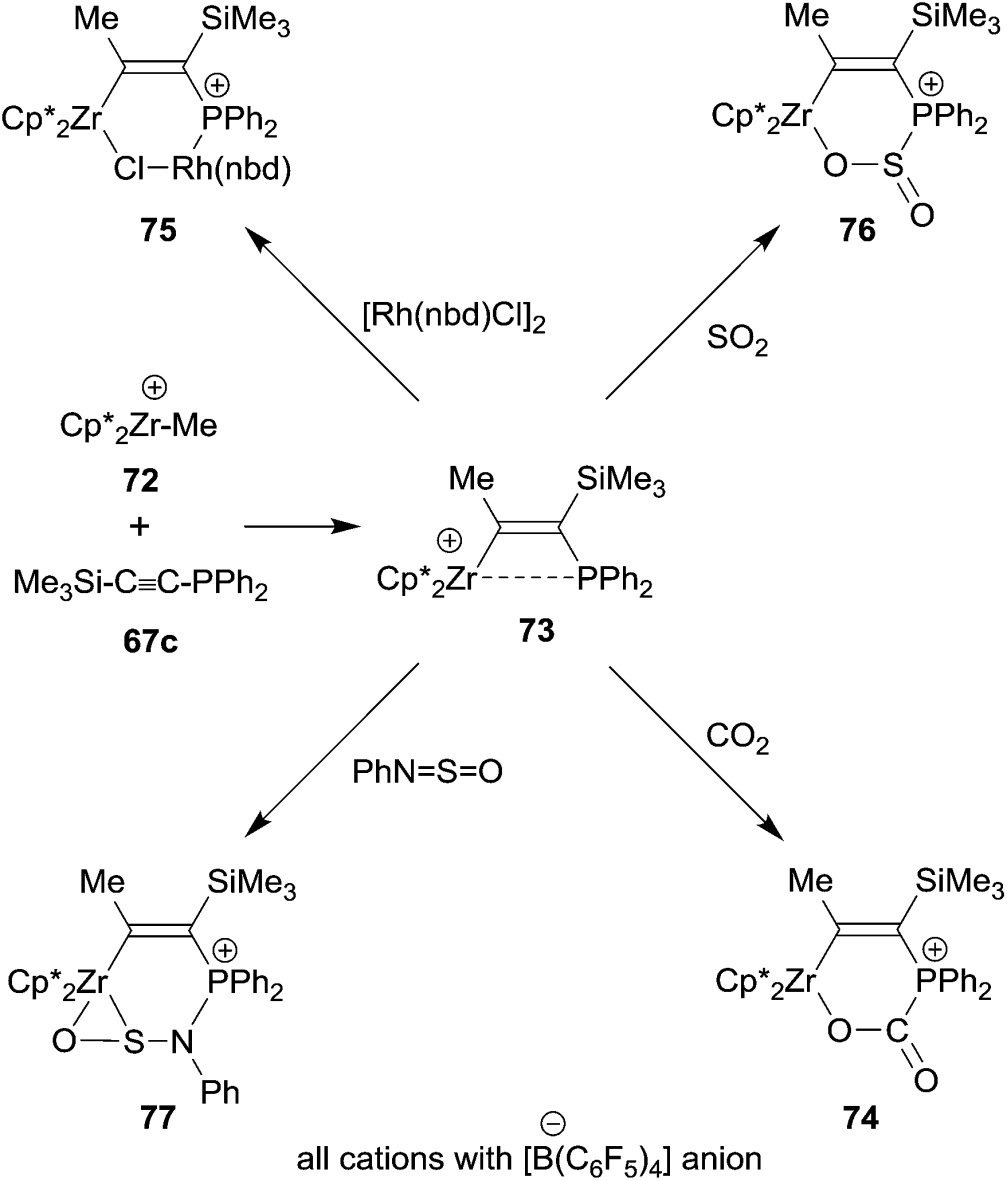


The general reaction scheme on which the 1,1-carboboration reaction is based has begun to stretch out to other areas. One is organometallic chemistry. Very reactive alkyl group 4 metallocene cations usually insert unsaturated organic substrates into their metal to carbon-σ-bond, *i.e.* they undergo a 1,2-carbometallation reaction. This reaction has provided the basis for the immensely important metallocene based homogeneous Ziegler–Natta olefin polymerization.^54^ We have recently found a deviation from this behaviour, namely the occurrence of a 1,1-carbozirconation reaction of a suitably substituted alkyne.^55^ We found that the bulky bis(pentamethylCp)ZrCH_3_^+^ cation **72** undergoes a clean 1,1-carbozirconation reaction with diphenylphosphinotrimethylsilylacetylene **67c** to give the bulky β-phosphanyl substituted alkenyl zirconium cation product **73** (see Scheme 22). Complex **73** undergoes a variety of typical frustrated Lewis pair addition reactions to unsaturated substrates. The Zr^+^/P FLP adds to organic carbonyl compounds, including carbon dioxide (**74**). It even adds to some transition metal complexes (**75**), thereby taking the role of an ambiphilic ligand.^56^ It even adds to SO_2_ giving the heterocyclic product **76**. With the related *N*-sulfinyl benzene amine reagent PhN=S=O compound **73** gave the unusually structured η^2^-O,S-addition product **77**.^55^

**Scheme 23:**
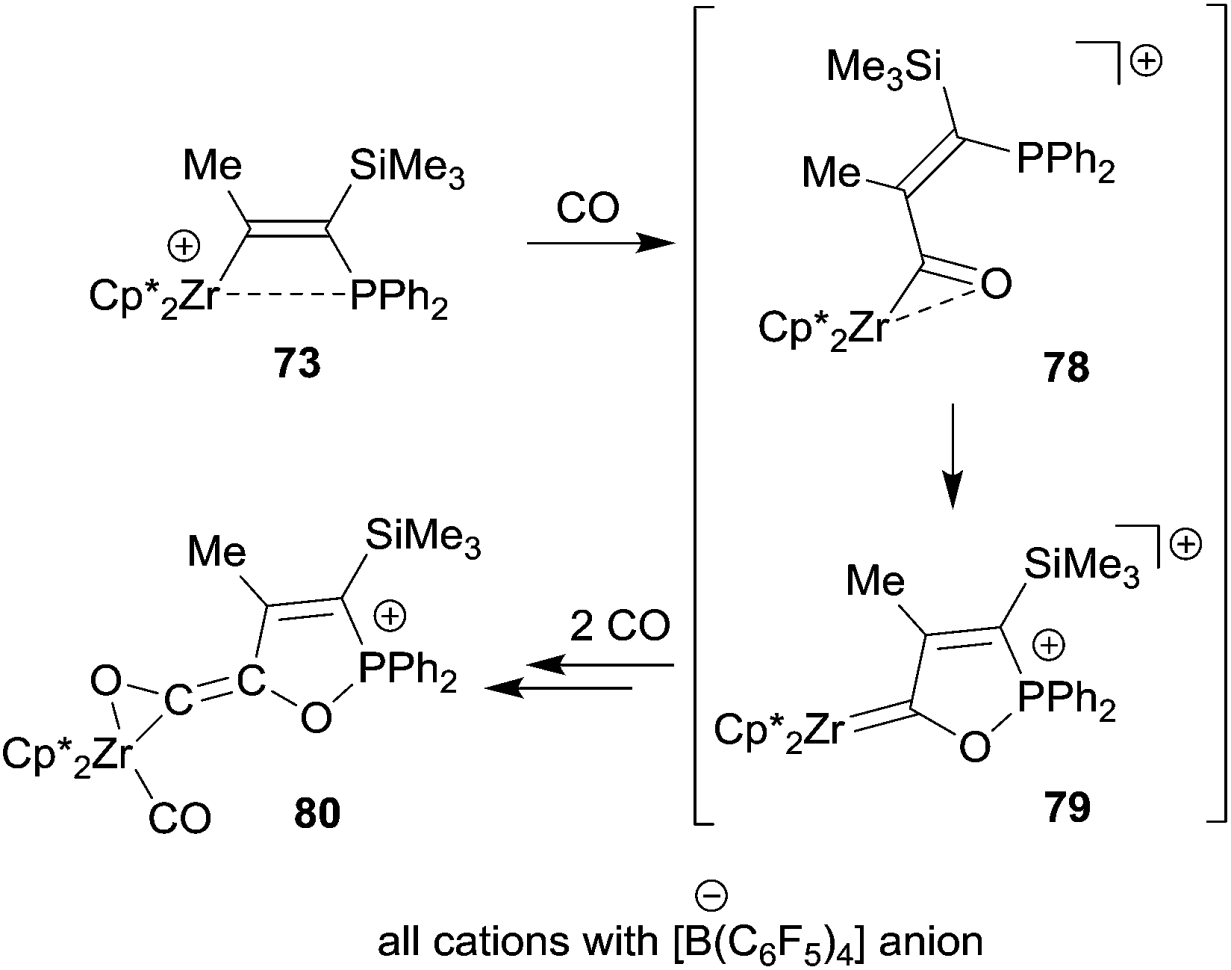


The Zr/P FLP **73** takes up three molar equivalents of carbon monoxide to give the (η^2^-ketene)zirconium carbonyl cation product **80**. We assume that this reaction is initiated by CO insertion into the metal carbon bond followed by cooperative activation of the acyl moiety for further CO uptake to eventually give the product **80** (see Scheme 23). Compound **73** was also shown to be a good catalyst for the selective head to tail dimerization of phenylacetylene.^55^

## Conclusions

1,1-Carboboration chemistry had started with the work by Binger, Köster and their contemporaries on the reactions of the alkynylborate anions with electrophiles. However, the finding, development and many applications of the neutral 1,1-carboboration reactions and the establishment of this chemistry as a new method for synthesizing alkenylboranes is due to Wrackmeyer's seminal work. Wrackmeyer *et al.* have almost single handed developed the by now classical procedures of converting alkynes with suitable metal-containing migrating groups to often useful alkenylboranes by reacting them with *e.g.* triethylborane, triallylborane or similar simple borane reagents. This and the further development of this chemistry has surely justified the use of the term “Wrackmeyer reaction” for this general class of alkyne/borane transformations. They especially developed this into methods of carrying out sequential 1,1-carboboration reactions starting from geminal metal bis-acetylides or even oligo-alkynes to form various types of alkenylborane containing heterocycles, most noteworthy the five-membered siloles, stannoles, *etc.* The latter were most readily formed because of the superb migrating abilities of the organostannyl groups. Although the resulting tin compounds did not have a prime interest in themselves, they constituted valuable reagents for transmetallation and for transformation into other heterocyclic ring types.

The use of the strongly Lewis acidic, strongly electrophilic R-B(C_6_F_5_)_2_ reagents has marked another more recent breakthrough in 1,1-carboboration chemistry. The use of these readily available reagents has allowed to carry out 1,1-carboboration reactions under much milder conditions and, consequently, has removed its restriction to the metal containing migrating groups (although silyl groups are still key fragments used for practical reasons). This has allowed to perform 1,1-carboboration reactions with simple alkynes carrying just conventional organic substituents. Many terminal alkynes have now been used since hydride migration along the alkynyl framework is often facile, but even internal alkynes have been successfully submitted to the advanced 1,1-carboboration reaction scheme, here proceeding with carbon–carbon bond cleavage. The use of the reactive R-B(C_6_F_5_)_2_ reagents has led to the development of sequential reactions for benzannulation. Since sulphur, phosphorus and even boron containing groups are migrating well, this has resulted in the development of interesting heterocycle synthesis of thiophenes, phospholes, and new boroles. The alkenyl-B(C_6_F_5_)_2_ functionalities in many of these compounds have been found to serve as useful reagents in cross-coupling reactions giving the respective B(C_6_F_5_)_2_ free organic products. In some cases the bulky alkenyl-B(C_6_F_5_)_2_ products found a high interest in themselves, *e.g.* as Lewis acid components in frustrated Lewis pair chemistry. Overall is seems that with these recent developments the 1,1-carboboration reaction is becoming more and more a viable synthetic alternative to the ubiquitous hydroboration reaction of alkynes, especially in cases where regioselectivity and bulkiness of substituents is critical. We also note that the general scheme of the 1,1-carboboration reaction is beginning to reach out to other areas of chemistry, as our recent example of the newly found 1,1-carbozirconation reaction illustrates. We will see which interesting new developments in this dynamic and rapidly developing field is lying ahead of us.
